# Synthesis of 5α-Androstane-17-spiro-δ-lactones with a 3-Keto, 3-Hydroxy, 3-Spirocarbamate or 3-Spiromorpholinone as Inhibitors of 17β-Hydroxysteroid Dehydrogenases

**DOI:** 10.3390/molecules18010914

**Published:** 2013-01-11

**Authors:** Guy Bertrand Djigoué, Béatrice Tchédam Ngatcha, Jenny Roy, Donald Poirier

**Affiliations:** Laboratory of Medicinal Chemistry, CHU de Québec (CHUL)—Research Center and Faculty of Medicine, Laval University, 2705 Laurier Boulevard, Québec (Québec), G1V 4G2, Canada

**Keywords:** synthesis, steroids, spirolactone, enzyme inhibitor, 17β-hydroxysteroid dehydrogenase

## Abstract

We synthesized two series of androstane derivatives as inhibitors of type 3 and type 5 17β-hydroxysteroid dehydrogenases (17β-HSDs). In the first series, four monospiro derivatives at position C17 were prepared from androsterone (ADT) or *epi*-ADT. After the protection of the alcohol at C3, the C17-ketone was alkylated with the lithium acetylide of tetrahydro-2-(but-3-ynyl)-2-H-pyran, the triple bond was hydrogenated, the protecting groups hydrolysed and the alcohols oxidized to give the corresponding 3-keto-17-spiro-lactone derivative. The other three compounds were generated from this keto-lactone by reducing the ketone at C3, or by introducing one or two methyl groups. In the second series, two dispiro derivatives at C3 and C17 were prepared from *epi*-ADT. After introducing a spiro-δ-lactone at C17 and an oxirane at C3, an aminolysis of the oxirane with L-isoleucine methyl ester provided an amino alcohol, which was treated with triphosgene or sodium methylate to afford a carbamate- or a morpholinone-androstane derivative, respectively. These steroid derivatives inhibited 17β-HSD3 (14–88% at 1 μM; 46–94% at 10 μM) and 17β-HSD5 (54–73% at 0.3 μM; 91–92% at 3 μM). They did not produce any androgenic activity and did not bind steroid (androgen, estrogen, glucocorticoid and progestin) receptors, suggesting a good profile for prostate cancer therapy.

## 1. Introduction

**Figure 1:**
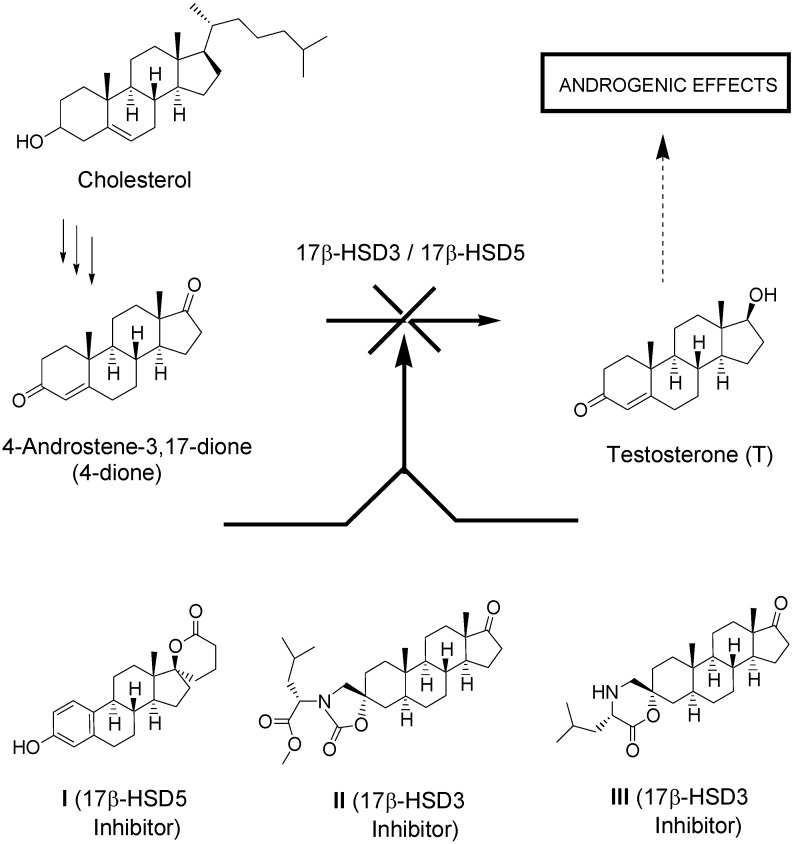
Role of 17β-HSD3 and 17β-HSD5 in the synthesis of the androgenic hormone testosterone.

Prostate cancer is an androgen-dependent disease that is well known for its high sensitivity to androgen deprivation. In fact, for over 50 years, the exclusive treatment of advanced metastatic prostate cancer was androgen deprivation achieved through castration, as it was believed that 95% of androgens were of testicular origin [1,2]. However, it is now well known that peripheral tissues represent another important source of androgens [3,4]. In fact, the prostatic tissues efficiently convert the hormone precursor dehydroepiandrosterone (DHEA) into the active androgens testosterone (T) and dihydrotestosterone (DHT) [5,6,7,8,9,10]. Both type 3 and type 5 17β-hydroxysteroid dehydrogenases (17β-HSD3 and 17β-HSD5, respectively) catalyse the reduction of 4-androstene-3,17-dione (4-dione) to testosterone (T) (Figure 1). However, whereas type 3 is located mainly in the testis, type 5 is expressed in the peripheral tissues [10]. In order to control the peripheral formation of active androgens, which could enhance the efficacy of endocrine therapy (such as the use of a pure antiandrogen with an LHRH agonist), we focused on the development of inhibitors of 17β-HSD3 and 17β-HSD5.

**Figure 2:**
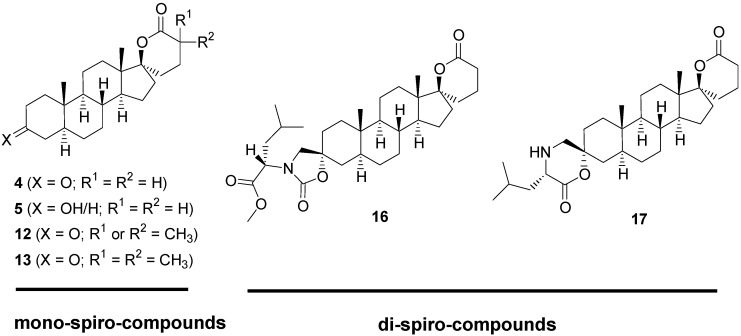
Chemical structures of potential inhibitors of 17β-HSD5 or 17β-HSD3 (monospiro-compounds: **4**, **5**, **12** and **13** and dispiro-compounds **16** and **17**).

Our group has previously demonstrated that a spiro-δ-lactone at position 17 and an estrane backbone are two important requirements for the inhibition of 17β-HSD5 [11]. We also reported that introducing a hydrophobic group at position 3 of androsterone (ADT) provides potent inhibitors of 17β-HSD3 [12,13,14,15,16]. For example, we recently published the inhibitory potency of 3-spiro-carbamate and 3-spiro-morpholinone ADT derivatives and their respective stereoisomers on 17β-HSD3 [17]. In our pursuit of the optimization of new 17β-HSD inhibitors, we synthesized steroid derivatives **4**, **5**, **12**, **13**, **16** and **17** (Figure 2). The monospiro derivatives **4**, **5**, **12** and **13** were designed to inhibit 17β-HSD5 whereas dispiro derivatives **16** and **17** were designed to inhibit 17β-HSD3. In this article, we report the synthesis, NMR characterization and biological activity of these new spiro derivatives (compounds **4**, **5**, **12**, **13**, **16** and **17**) (Figure 2).

## 2. Results and Discussion

### 2.1. Synthesis of Spiro-δ-Lactones ***4*** and ***5*** (Scheme 1)

The natural C19-steroid *epi*-androsterone (epi-ADT) was the starting material used in the synthesis of spiro-δ-lactones **4** and **5**. After protecting the 3β-OH as a tetrahydropyranyl (THP) ether using dihydropyran in the presence of a catalytic amount of *p*-toluenesulfonic acid (*p*-TSA), the carbonyl group at position 17 was alkylated with the lithium acetylide generated from 2-(3-butynyloxy)-tetrahydro-2*H*-pyran and *n*-BuLi. As reported in the literature, the acetylide attacks the carbonyl group by the less hindered alpha face of the steroid, providing the alkyne derivative **2** [18,19,20]. The triple bond of the alkylated steroid **2** was hydrogenated using a mixture of Pd/C and Pd/CaCO_3_, under a hydrogen atmosphere to yield the corresponding alkane **3**. Without purification, the di-THP derivative **3** was treated with Jones’ reagent leading to the spiro-δ-lactone **4**. Under these conditions, both deprotection and oxidation of secondary and primary alcohols occurred followed by lactonization between the 17β-OH and the carboxylic acid at the end of the side chain. Formation of the spiro-δ-lactone ring was confirmed by the presence of characteristic signals in ^13^C-NMR (93.21 and 171.98 ppm for C-17 and COO, respectively), which are identical to those of the corresponding spiro-δ-lactone with an estrane nucleus [20]. The carbonyl group of ketone **4** was reduced with NaBH_4_ in MeOH to yield **5** as an epimeric mixture of 3β- and 3α-alcohols (3β-OH/3α-OH: 85/15). The major 3β-OH product was identified by the 3α-CH signal at 3.55 ppm in ^1^H NMR whereas the 3β-CH of the minor 3α-OH product appears at 4.01 ppm. These two signals are similar to those obtained from commercially available samples of *epi*-ADT (3α-CH: 3.60 ppm) and ADT (3β-CH: 4.07 ppm).

**Scheme 1:**
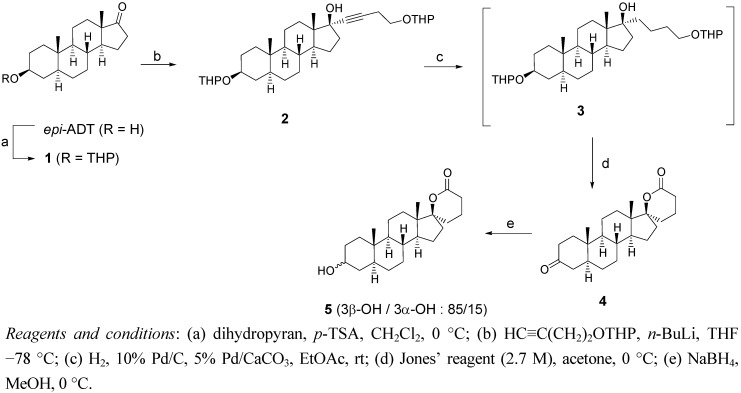
Synthesis of spiro-δ-lactones **4** and **5**.

### 2.2. Synthesis of Methylated Spiro-δ-Lactones ***12*** and ***13*** (Scheme 2)

**Scheme 2:**
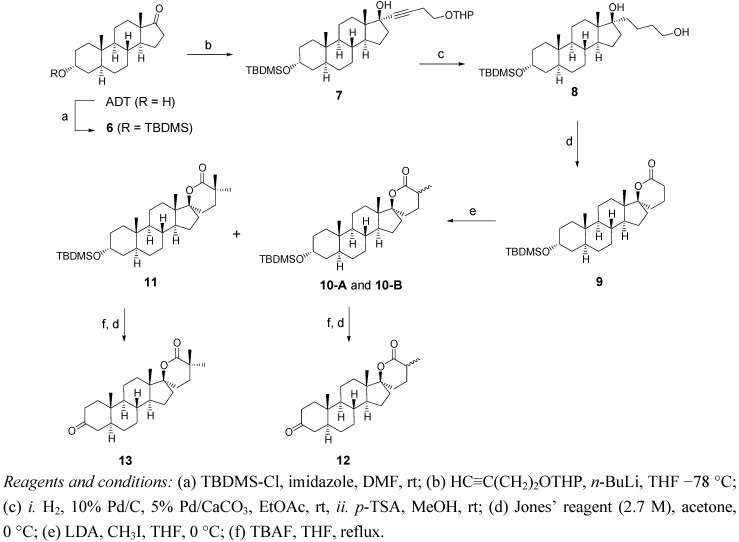
Synthesis of methylated spiro-δ-lactones **12** and **13**.

The monomethylated and dimethylated spiro-δ-lactones **12** and **13** were obtained from androsterone (ADT) through the sequence of reactions depicted in Scheme 2. ADT was first protected as a silylated ether using *tert*-butyldimethylsilyl-chloride (TBDMS-Cl) and imidazole in DMF. The anion resulting from the reaction between *n*-BuLi and 2-(3-butynyloxy)tetrahydro-2*H*-pyran was used for the alkylation of the carbonyl of TBDMS-ADT (**6**). The alkyne **7** was submitted to hydrogenation conditions (H_2_, Pd/C and Pd/CaCO_3_) to yield the corresponding alkane, which was treated *insitu* with *p*-TSA in MeOH at room temperature to selectively hydrolyze the THP group. The diol **8** was then treated with Jones' reagent to yield the spiro-δ-lactone **9**. Alkylation in α-position of the lactone carbonyl was performed with lithium diisopropylamide (LDA) and methyl iodide. A mixture of three α-methylated lactones was obtained: the dimethylated lactone **11** and the two possible monomethylated spiro-δ-lactones **10A** and **10B**. Methylated spiro-lactones **10A**, **10B** and **11** were oxidized with Jones’ reagent leading to compounds **12** and **13**. Unfortunately, epimerisation at position α of the lactone occurred when submitting each of the monomethylated compounds **10A** and **10B** to hydrolysis and oxidative conditions. The same mixture of the two possible monomethylated compounds was thus obtained, and this time, it was not possible to separate them by column chromatography.

### 2.3. Synthesis of Spiro-Carbamate ***16*** and Spiro-Morpholinone ***17*** (Scheme 3)

Following a slightly modified method reported previously in our laboratory [21], the 3-oxo-spiro-δ-lactone **4** was reacted with trimethylsulfoxonium iodide (four equivalents rather than two) to yield oxirane **14**. The 3β-CH_2_ orientation of **14** was confirmed by a correlation with methyl 19 in NOESY spectrum. Compound **14** was then submitted to an aminolysis with L-leucine methyl ester to generate the amino alcohol **15**. The amino group of L-leucine methyl ester was previously generated from the commercially available chlorhydrate [21]. The spiro-carbamate **16** was obtained from the reaction of **15** with triphosgene. Because the reaction was very slow with only 0.5 equivalent of triphosgene, the quantity reported to produce a similar carbamate [14], we used one equivalent to complete the reaction generating **16**. The formation of a carbamate group was confirmed by a characteristic signal at 157.64 ppm in ^13^C-NMR. The spiro-morpholinone **17** was obtained from the lactonization of the amino alcohol **15**. During this step, unknown products are formed; this explains the poor yield of this reaction. This is probably due to the polymerization of the starting amino alcohol or the partial aminolysis of the spiro-δ-lactone. HPLC chromatogram showed four resolved peaks, integrating for 39% (expected compound **17**), 18% (the starting amino alcohol **15**) and two other peaks representing unknown products. The formation of the spiro-morpholinone moiety was confirmed by a characteristic signal at 171.99 ppm in ^13^C-NMR.

**Scheme 3:**
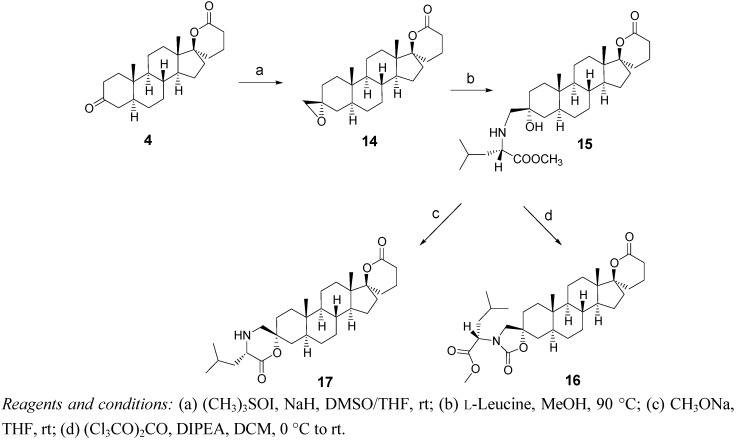
Synthesis of dispirolactone **16** (carbamate) and spiromorpholinone **17**.

### 2.4. Biological Evaluation of Monospiro Derivatives ***4***, ***5***, ***12*** and ***13***

Compounds **4**, **5**, **12** and **13** were evaluated for their ability to inhibit the 17β-HSD5 activity found in transfected HEK-293 cells by measuring the amount of labelled testosterone (T) formed from labelled natural substrate 4-dione (Table 1). All compounds inhibited the 17β-HSD5 (91–92% at 3 μM), but the C19-steroid (androstane) backbone seem to be less efficient than the C18-steroid (estrane) backbone. In fact, the androstane spiro-δ-lactone **4** is a less potent inhibitor than the corresponding estrane compound **I** (64 and 92% of inhibition at 0.3 µM, respectively). The monomethylation of lactone **4** (compound **12**) brought a slight increase in the inhibitory activity (73% at 0.3 µM), whereas the dimethylation (compound **13**) brought a small decrease of inhibition (54% at 0.3 µM). The spiro-δ-lactone bearing a hydroxyl at position 3, compound **5**, gave a 56% inhibition of 17β-HSD5 at 0.3 µM, a value lower than that of the corresponding keto compound **4** (64% of inhibition). Compound **4**, **5**, **12** and **13** were also tested as inhibitor of 17β-HSD3 by measuring the transformation of labelled 4-dione to labelled T by a microsomal preparation of rat testis. No significant inhibition was observed at concentrations of 0.1 and 1 μM for all compounds, and only small inhibitory activities (46–58%) were obtained at the higher concentration of 10 μM. This is fully in accord with our first structure-activity relationship (SAR) results that identified the importance of a hydrophobic group at position 3 of ADT, instead of at position 16, to inhibit 17β-HSD3 [13,16].

After we determined the inhibitory potency of a spiro-δ-lactone at position 17 of an androstane backbone on 17β-HSD5 and 17β-HSD3, we evaluated their proliferative activity on Shionogi androgen-sensitive cell lines. In fact, for a potential use in prostate cancer, an enzyme inhibitor should be devoid of proliferative androgenic activity. The behaviour of compounds **4**, **5**, **12** and **13** on an androgen-sensitive Shionogi cell line was then evaluated and compared to that of hydroxyflutamide, a well known antiandrogen (Table 2) [22,23].

**Table 1:** Inhibition of 17β-HSD5 and 17β-HSD3 by compounds **4**, **5**, **12**, **13**, **16** and **17**.

Compounds (characteristics) *^a^*	Inhibition of 17β-HSD5 at 0.3 μM (%) *^b^*	Inhibition of 17β-HSD5 at 3 μM (%) *^b^*	Inhibition of 17β-HSD3 at 0.1 μM (%) *^c^*	Inhibition of 17β-HSD3 at 1 μM (%) *^c^*	Inhibition of 17β-HSD3 at 10 μM (%) *^c^*
**I** (C18/17-lactone/3-OH	92	95	--	--	--
**II** (C19/17-oxo/3-carbamate	--	--	66.0 ± 1.7	88.3 ± 1.1	93.7 ± 0.8
**III** (C19/17-oxo/3-morpholinone	--	--	63.2 ± 2.6	81.0 ± 1.6	88.7 ± 4.0
**4** (C19/17-lactone/3-oxo	64	92	4.9 ± 4.8	15.0 ± 0.9	56.7 ± 2.2
**5** (C19/17-lactone/3-OH	56	91	0.6 ± 5.3	22.4 ± 2.8	45.7 ± 1.5
**12** (C19/17-lactone; mono-CH_3_/3-oxo	73	91	1.0 ± 3.3	22.2 ± 4.9	53.4 ± 5.0
**13** (C19/17-lactone; bis-CH_3_/3-oxo	54	91	1.0 ± 7.2	14.4 ± 3.3	58.0 ± 3
**16** (C19/17-lactone/3-carbamate	--	--	32.0 ± 3.3	60.4 ± 4.4	60.9 ± 0.7
**17** (C19/17-lactone/3-morpholinone	--	--	11.2 ± 2.6	51.0 ± 1.5	87.2 ± 0.5

*^a^* C18: estrane nucleus (18 carbons) and C19: androstane nucleus (19 carbones); *^b^* For the transformation of [^14^C]-4-dione to [^14^C]-T by HEK-293 cells overexpressing human 17β-HSD5 (transfected cells in culture); *^c^* For the transformation of [^14^C]-4-dione to [^14^C]-T by rat testicular 17β-HSD3 (microsomal fraction).

**Table 2:** Proliferative an antiproliferative activities of monospiro-compounds **4**, **5**, **12** and **13** on Shionogi (AR^+^) cells.

Compounds	Proliferative activity at 0.1 μM (%) *^a^*	Proliferative activity at 1 μM (%) *^a^*	Antiproliferative activity at 0.1 μM (%) *^b^*	Antiproliferative activity at 1 μM (%) *^b^*
**4**	12 ± 2	0 ± 5	23 ± 1	100 ± 1
**5**	19 ± 4	0 ± 5	10 ± 1	61 ± 3
**12**	9 ± 5	0 ± 3	0 ± 5	58 ± 2
**13**	0 ± 9	0 ± 5	1 ± 2	49 ± 7
**OH-Flu *^c^***	0 ± 6	0 ± 2	69 ± 3	100 ± 3

*^a^* The proliferative activity expressed in percentage was calculated in comparison to the stimulation (100%) induced by 0.3 nM of potent androgen dihydrotestosterone (DHT); *^b^* The antiproliferative activity expressed in percentage is the ability of a compound to inhibit the 0.3 nM DHT-induced proliferation of cells; *^c^* The antiandrogen hydroxyflutamide was used as a reference compound [22,23].

Except for the α-dimethylated lactone **13** which did not show any proliferative (androgenic) activity, the C19-steroid spiro-δ-lactones **4**, **5** and **12** exhibited a slight proliferative activity at 0.1 μM on Shionogi cells (12, 19 and 9%, respectively). However, at the higher concentration of 1 μM, no proliferative activity was observed for spiro-δ-lactones **4**, **5**, **12** and **13** suggesting that proliferative effects observed at 0.1 μM were not significant. The antiproliferative (antiandrogenic) activity was measured by the inhibition of DHT (0.3 nM)-induced proliferation on Shionogi cells. All the target compounds **4**, **5**, **12** and **13** showed an antiproliferative activity at 1 μM, the most important effect being observed with the spiro-δ-lactone **4** (100% antiproliferative activity). Its activity dropped down at lower concentration: 23% at 0.1 μM, compared to 69% for hydroxyflutamide at the same concentration.

To discriminate between two possible antiproliferative effects: an antiandrogenic activity mediated by the androgen receptor (AR) and a cytotoxic activity not mediated by AR, we measured the binding affinity on AR for each compound (Table 3). In fact, the C19-steroids **4**, **5**, **12** and **13** were expected to show some affinities with androgen receptor, as their chemical structures are similar to that of the natural substrate T and DHT. Compounds **4**, **5** and **12** showed a weak binding on AR suggesting an antiandrogenic effect instead of a cytotoxic effect. It is however possible that the antiproliferative effect we have observed on Shionogi cells was a mixture of both antiandrogenic and cytotoxic activities. Interestingly, the spiro-lactones **4**, **5**, **12** and **13** did not show affinities with other steroid (estrogen, glucocorticoid and progestin) receptors.

**Table 3:** Binding affinity (%) of compounds **4**, **5**, **12** and **13** on steroid receptors.

**Cpds**	**Androgen receptor (%) *^a^***		**Estrogen receptor-α (%) *^a^***		**Glucocorticoid receptor (%) *^a^***		**Progestin receptor (%) *^a^***	
	10 nM	1 µM	10 nM	1 µM	10 nM	1 µM	10 nM	1 µM
**4**	4 ± 1	9 ± 2	0 ± 1	1 ± 1	0 ± 3	0 ± 1	4 ± 2	0 ± 2
**5**	7 ± 1	5 ± 1	0 ± 1	0 ± 1	0 ± 1	1 ± 2	4 ± 1	1 ± 2
**12**	1 ± 2	4 ± 2	0 ± 2	1 ± 3	0 ± 3	0 ± 1	0 ± 2	0 ± 1
**13**	0 ± 2	0 ± 2	0 ± 2	0 ± 3	0 ± 2	1 ± 3	0 ± 3	1 ± 3
DHT *^b^*	70 ± 1	100 ± 1	2 ± 2	4 ± 1	2 ± 2	6 ±2	3 ± 2	40 ± 2
E2 *^b^*	0 ± 2	34 ± 1	75 ± 1	100 ± 1	5 ± 2	12 ± 2	6 ± 3	25 ± 2
DEX *^b^*	0 ± 1	2 ± 1	0 ± 3	0 ± 1	66 ± 2	99 ± 1	0 ± 3	1 ± 2
R5050 *^b^*	1 ± 4	28 ± 2	5 ± 2	4 ± 1	9 ± 2	85 ± 2	65 ± 2	99 ± 2

*^a^* Data are expressed in percentage (%) of the binding affinity obtained with a natural or synthetic ligand tested at a concentration of 1 μM (100% of binding); *^b^* Natural and synthetic ligands used for each receptor: DHT, dihydrotestosterone; E2, estradiol; DEX, dexamethasone; R5050, synthetic progestin.

### 2.5. Biological Evaluation of Dispiro Derivatives ***16*** and ***17***

Compounds **16** and **17** were synthesized to determine the impact of two spiro-functionalities on the inhibition of 17β-HSD3 (Table 1). As mentioned above, a microsomal preparation of rat testis was used as source of enzyme activity transforming 4-dione to T. In the first series of monospiro derivatives (compounds **4**, **5**, **12** and **13**), the presence of a spiro-δ-lactone at position C-17 resulted in a very weak inhibition (1–5% at 0.1 μM). At the opposite, the presence of a carbamate or a morpholinone moiety at position C-3 generated a very good inhibitory activity (66 and 63% at 0.1 μM) for monospiro derivatives II and III, respectively. When we introduced a carbamate or a morpholinone at position C-3 of compound **4**, both dispiro derivatives **16** and **17** produced a moderate inhibitions of 17β-HSD3 (32 and 11% at 0.1 μM; 60 and 51% at 1 μM), which are less important than those of known inhibitors II and III (only C-3 derivatives) and more important than those of **4** and **5** (only C-17 derivatives).

## 3. Experimental

### 3.1. General

Chemical reagents as well as DMF and CH_2_Cl_2_, 99.8% anhydrous grade, were purchased from Aldrich Chemical Company (Milwaukee, WI, USA). Androsterone and *epi*-androsterone were obtained from Steraloids (Wilton, NH, USA). THF, used in anhydrous conditions, was distilled from sodium benzophenone ketyl. Solvents for chromatographies were purchased from BDH Chemicals (Montréal, QC, Canada) or Fisher Chemicals (Montréal, QC, Canada). Thin-layer chromatography (TLC) was performed on 0.20 mm silica gel 60 F_254_ plates (E. Merck, Darmstadt, Germany) and 230–400 mesh ASTM silica gel 60 (E. Merck) was used for flash chromatography. Infrared spectra (IR) are reported in cm^−1^ and obtained on a Perkin-Elmer 1600 (FT-IR series) spectrophotometer. Nuclear magnetic resonance spectra (NMR) were recorded with a Bruker AC/F 300 spectrometer (Billerica, MA, USA) at 300 (^1^H) and 75 (^13^C) MHz or a Bruker AVANCE 400 spectrometer at 400 (^1^H) and 100 (^13^C) MHz. The chemical shifts (δ) are expressed in ppm and referenced to chloroform (7.26 ppm for ^1^H and 77.00 ppm for ^13^C). All ^13^C-NMR signals of final compounds **4**, **5**, **12**, **13**, **16** and **17** (Table 4) were fully assigned using a series of NMR experiments (APT, HSQC, HMBC, COSY and NOESY) and data reported in literature [20,21,24,25,26,27]. High-resolution mass spectra (HRMS) were provided by Pierre Audet at the Laval University Chemistry Department (Québec, QC, Canada). The names of steroid derivatives were generated using ACD/Labs (Chemist’version) software (Toronto, ON, Canada).

**Table 4:** ^13^C-NMR data of final compounds **4**, **5**, **12**, **13**, **16** and **17** dissolved in CDCl_3_. 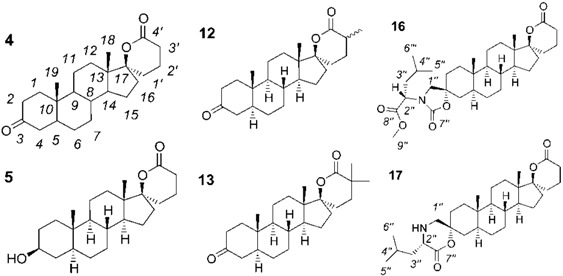

Cpds	4	5		12		13		16		17	
**C1**	38.55	36.98		38.60		38.58		33.86		32.98	
**C2**	38.07	31.38		38.12		38.11		32.75		31.48	
**C3**	211.67	71.07		211.79		211.84		79.74		82.37	
**C4**	44.59	38.03		44.63		44.62		39.48		38.11	
**C5**	46.71	44.84		46.75		46.74		40.86		39.29	
**C6**	28.72	28.47		28.77		28.75		28.01		28.00	
**C7**	31.40	31.72		31.44		31.41		31.47		31.38	
**C8**	35.75	35.82		35.81		35.81		35.84		35.85	
**C9**	53.61	54.12		53.67		53.63		53.61		53.50	
**C10**	35.75	35.49		35.81		35.81		35.31		36.02	
**C11**	20.90	20.65		20.91 (20.98)		20.97		20.43		20.46	
**C12**	31.86	31.92		31.93		31.92		31.93		31.96	
**C13**	46.99	46.95		46.96		47.01		46.99		46.99	
**C14**	49.64	49.77		49.45 (49.61)		49.49		49.77		49.83	
**C15**	23.77	23.73		23.94 (23.72)		23.61		23.76		23.78	
**C16**	33.93	33.90		33.99 (33.50)		34.77		33.95		33.92	
**C17**	93.21	93.34		92.78 (93.65)		93.70		93.40		93.42	
**C18**	14.40	14.37		14.52		14.56		14.43		14.45	
**C19**	11.43	12.24		11.47		11.45		11.40		11.36	
**C1'**	27.86	27.82		27.21 (28.58)		25.51		27.91		27.89	
**C2'**	15.83	15.78		24.41 (24.21)		31.55		15.84		15.84	
**C3'**	29.40	29.37		34.64 (36.16)		37.76		29.47		29.47	
**C4'**	171.98	172.13		174.90 (175.89)		177.90		172.22		172.25	
**C5’**	---	---		17.35 (17.26)		27.65		---		---	
**C6'**	---	---		---		27.75		---		---	
**C1''**	---	---		---		---		52.89		52.41	
**C2''**	---	---		---		---		53.61		55.58	
**C3''**	---	---		---		---		37.69		41.41	
**C4''**	---	---		---		---		24.93		24.46	
**C5''/C6''**	---	---		---		---		21.06/23.14		20.97/23.40	
**C7''**	---	---		---		---		157.99		171.99	
**C8''**	---	---		---		---		171.99		---	
**C9''**	---	---		---		---		52.22		---	

### 3.2. Synthesis of δ-Lactones ***4*** and ***5*** (Scheme 1)

*(3β,5α)-3-(Tetrahydro-2H-pyran-2-yloxy)androstan-17-one* (**1**). To a solution of *epi*-androsterone (2.0 g, 6.89 mmol) in dry CH_2_Cl_2_ (150 mL) was added 3,4-dihydropyrane (1.9 mL, 3 eq) and *p*-toluene-sulfonic acid (0.130 g, 0.1 eq). The mixture was stirred for 2 h at 0 °C under an atmosphere of argon. The reaction was stopped by adding a saturated NaHCO_3_ solution. Extraction was done with EtOAc, the organic layer was washed with water, then dried over MgSO_4_ and evaporated to dryness under reduced pressure. The yellow oil obtained was purified by flash chromatography, using a mixture of hexanes and EtOAc (8/2) as eluent, to give the compound **1** as a white amorphous solid in 82% yield. IR (film) υ 1740 (C=O, ketone); ^1^H-NMR (CDCl_3_) δ 0.83 and 0.85 (2s, CH_3_-18 and CH_3_-19), 0.60–2.10 (unresolved CH and CH_2_), 2.43 (dd, *J*_1_ = 19.0 Hz and *J*_2_ = 8.8 Hz, CH-16β), 3.48 and 3.92 (2m, CH_2_O of THP), 3.58 (m, CH-3α), 4.71 (t, *J* = 3.7 Hz, CH of THP); ^13^C-NMR (CDCl_3_) δ 12.28, 13.81, 20.04, 20.49, 21.78, 25.52, 27.79, 28.42, (28.56), 29.41, (29.70), 30.94, 31.30, 31.59, 34.29, 35.09, 35.85, (36.15), 36.96, (37.17), 44.80, (45.14), 47.80, 51.46, 54.52, 62.81, 75.09, (75.45), 96.60, (96.96), 221.32.

*17β-Hydroxy-3β-(tetrahydro-2H-pyran-2-yloxy)-17α-[4'-(tetrahydro-2H-pyran-2-yloxy)butynyl]-5α-androstane* (**2**). To a solution of 2-(3-butynyloxy)-tetrahydro-2*H*-pyrane (3.1 mL, 19.86 mmol) in dry THF (100 mL) under an argon atmosphere was added a 1.6 M solution of *n*-BuLi in hexanes (12.4 mL, 19.65 mmol) and the reaction mixture was stirred at −78 °C for 20 min. A solution of ketone **1** (1.85 g, 4.95 mmol) in dry THF (50 mL) was then added dropwise over a period of 15 min and the mixture was allowed to react overnight, the temperature going from −78 °C to room temperature. A saturated NaHCO_3_ solution was added and the extraction was done with EtOAc. The organic layer was washed with brine, dried over MgSO_4_ and evaporated to dryness under reduced pressure. The crude product was purified by column chromatography using a mixture of hexanes and EtOAc (8/2) as eluent to give the alkylated compound **2** as an amorphous white solid in 68% yield. IR (film) υ 3429 (OH, alcohol); ^1^H-NMR (CDCl_3_) δ 0.76 and 0.77 (2s, CH_3_-18 and CH_3_-19), 0.55–2.20 (unresolved CH and CH_2_), 2.49 (t, *J* = 6.9 Hz, C≡CCH_2_), 3.50 and 3.84 (2m, 2 × CH_2_O of THPs), 3.75 (m, CH-3α), 4.63 and 4.68 (2t, *J* = 3.1 Hz, 2 × CH of THPs); ^13^C-NMR (CDCl_3_) δ 12.18, 12.79, 19.13, 19.79, 19.85, 20.25, 20.79, 23.04, 25.36, 25.41, 27.70, 28.50, (28.65), 29.31, 30.44, 31.16, 31.50, 32.72, 34.22, 35.65, (36.06), 36.85, (37.07), 38.94, 44.66, (45.01), 46.78, 50.24, 53.90, 61.79, 62.49, (62.59), 65.71, 75.08, (75.41), 79.75, 82.76, 84.73, 96.37, (96.64), 98.43.

*(5S,8R,9S,10S,13S,14S,17S)-10,13-Dimethylhexadecahydrospiro[cyclopenta[a]phenanthrene-17,2'-pyran]-3,6'(2H,3'H)-dione* (**4**). To a solution of alkyne **2** (1.5 g, 2.94 mmol) in EtOAc (100 mL) was added a 1:1 mixture of palladium on charcoal (10%) and palladium on calcium carbonate (5%) (150 mg). The reaction mixture was stirred overnight at room temperature under an atmosphere of hydrogen. The mixture was filtered through a pad of celite and the solvent evaporated to dryness under reduced pressure. Without purification, the white solid corresponding to hydrogenated compound **3** was directly used for the next step. To a stirred solution of **3** (1.4 g) in acetone (100 mL) stirred at 0 °C was added dropwise a 2.7 M solution of Jones' reagent (3.5 mL). The reaction was monitored by TLC and was completed after 30 min. Isopropyl alcohol was then added until a persistent green colour remained. Organic solvents were removed under reduced pressure and the resulting green concentrate dissolved in water. Extraction was done with EtOAc and the combined organic layers were washed with brine, dried over MgSO_4_ and evaporated to dryness. Purification by column chromatography using a mixture of hexanes and EtOAc (9/1) gave the spiro-δ-lactone **4** as white solid in 98% yield (for the two steps). IR (film) υ 1714 (C=O, ketone and lactone); ^1^H-NMR (CDCl_3_) δ 0.98 (s, CH_3_-19), 1.02 (s, CH_3_-18), 0.60–2.58 (unresolved CH and CH_2_); ^13^C-NMR (CDCl_3_) δ 11.43, 14.40, 15.83, 20.90, 23.77, 27.86, 28.72, 29.40, 31.40, 31.86, 33.93, 35.75 (2×), 38.07, 38.55, 44.59, 46.71, 46.99, 49.64, 53.61, 93.21, 171.98, 211.67; HRMS for C_23_H_38_NO_3_ [M+NH_4_]^+^: calculated 376.2846, found 376.2850.

*(3S,5S,8R,9S,10S,13S,14S,17S)-3-Hydroxy-10,13-dimethyloctadecahydrospiro[cyclopenta[a]phenanthrene-17,2'-pyran]-6'(3'H)-one* (**5**). Ketone **4** (200 mg, 0.549 mmol) was dissolved in MeOH (30 mL) and NaBH_4_ (23 mg, 1.1 eq) was added. The solution was stirred at room temperature for 3 h. Water was then added, MeOH was evaporated under reduced pressure and the product was extracted with EtOAc. The organic layer was dried over MgSO_4_, the solvent evaporated under reduced pressure and the crude product was purified by column chromatography using a mixture of hexanes and EtOAc (8/2) to give the alcohol **5** as an epimeric mixture at position 3 (3β-OH/3α-OH: 85/15, evaluated by ^1^H-NMR). White solid (85% yield); IR (film) υ 3418 (OH, alcohol), 1716 (C=O, lactone); ^1^H-NMR (CDCl_3_) δ 0.77 and 0.79 (2s, CH_3_-19 of both epimers), 0.93 (s, CH_3_-18), 0.50–2.00 (unresolved CH and CH_2_), 2.43 (m, CH_2_COO), 3.55 and 4.01 (2m, CH-3β and CH-3α in proportions 85/15); ^13^C-NMR (CDCl_3_) *minor compound signals indicated between* [ ] δ [11.10], 12.24, 14.37, 15.78, 20.65, 23.73, 27.82, 28.47, 29.37, [29.61], 31.38, 31.72, 31.92, [32.15], 33.90, 35.49, 35.82, [36.08], 36.98, 38.03, [39.05], 44.84, 46.95, 49.77, 54.12, [66.31], 71.07, 93.34, 172.13; HRMS for C_23_H_40_NO_3_ [M+NH_4_]^+^: calculated 378.3003, found 378.3008.

### 3.3. Synthesis of α-Methylated δ-lactones 12 and 13 (Scheme 2)

(*3α,5α)-3-{[tert-Butyl(dimethyl)silyl]oxy}androstan-17-one* (**6**). The hydroxy group of androsterone (1.5 g, 5.17 mmol) was protected as a silylated ether in a mixture of dry DMF (100 mL), imidazole (1.76 g, 5 eq) and TBDMS-Cl (2.34 g, 3 eq). TBDMS-ADT (**6**) was thus obtained as a white solid in 94% yield and the IR, NMR and MS data are in accord with those reported in literature [28].

*3α-(tert-Butyldimethylsilyloxy)-17β-hydroxy-17α-[4'-(tetrahydro-2H-pyran-2-yloxy)butynyl]-5α-androstane* (**7**). The carbonyl group of TBDMS-ADT (**6**) was alkylated with 2-(3-butynyloxy)tetrahydro-2*H*-pyran as described above for the synthesis of compound **2**. Compound **7** was obtained as colourless oil in 70% yield. IR (film) υ 3444 (OH, alcohol); ^1^H-NMR (CDCl_3_) δ 0.02 (s, Si(CH_3_)_2_), 0.76 (s, CH_3_-19), 0.81 (s, CH_3_-18), 0.89 (s, SiC(CH_3_)_3_), 0.70–2.25 (unresolved CH and CH_2_), 2.55 (t, *J* = 6.8 Hz, C≡CCH_2_), 3.55 and 3.84 (2m, 2 × CH_2_O of THP and side chain), 3.95 (t, *J* = 2.1 Hz, CH-3β), 4.68 (t, *J* = 3.0 Hz, CH of THP); ^13^C-NMR (CDCl_3_) δ −4.85 (2×), 11.40, 12.90, 18.12, 19.26, 20.44, 23.15, 25.49, 25.63, 25.89 (3×), 28.59, 29.72, 30.57, 31.62, 32.35, 32.83, 36.09, 36.22, 36.78, 39.06, 46.24, 46.93, 50.37, 53.87, 61.92, 65.81, 66.86, 80.11, 83.16, 84.69, 98.62.

*3α-(tert-Butyldimethylsilyloxy)-17β-hydroxy-17α-(4'-hydroxybutyl)-5α-androstane* (**8**). Compound **7** was submitted to hydrogenation conditions as described in the first part of the synthesis of **4**. The crude product was then used without purification for the next step, the hydrolysis of the THP group. The crude hydrogenated product (3.6 g, 6.406 mmol) was dissolved in MeOH and *p*-TSA (122 mg, 0.1 eq) was added. The reaction mixture was stirred at room temperature for 1 h. Water was added, the MeOH evaporated under reduced pressure and the mixture extracted with EtOAc. The organic layer was washed with brine, dried over MgSO_4_ and evaporated to dryness. The crude product was purified by column chromatography using a mixture of hexanes and EtOAc (5/5) as eluent to give the diol **8** as a white solid in 80% (for the two steps). IR (film) υ 3351 (OH, alcohol); ^1^H-NMR (CDCl_3_) δ 0.01 (s, Si(CH_3_)_2_), 0.77 (s, CH_3_-19), 0.85 (s, CH_3_-18), 0.89 (s, SiC(CH_3_)_3_), 0.62–2.05 (unresolved CH and CH_2_), 3.69 (m, CH_2_OH), 3.95 (t, *J* = 2.2 Hz, CH-3β); ^13^C-NMR (CDCl_3_) δ −4.86 (2×), 11.41, 14.41, 18.10, 19.80, 20.45, 23.65, 25.86 (3×), 28.56, 29.72, 31.61, 31.90, 32.43, 33.41, 34.51, 36.04, 36.16, 36.41, 36.74, 39.08, 46.45, 50.53, 54.36, 62.93, 66.83, 83.59.

*(3S,5S,8R,9S,10S,13S,14S,17S)-3-{[tert-Butyl(dimethyl)silyl]oxy}-10,13-dimethyloctadecahydrospiro[cyclopenta[a]phenanthrene-17,2'-pyran]-6'(3'H)-one* (**9**). The diol **8** was oxidized with Jones' reagent as described in the second part of the synthesis of **4**. After purification by column chromatography using a mixture of hexanes and EtOAc (8/2) as eluent, lactone **9** was obtained in 95% yield as a white solid. IR (film) υ 1734 (C=O, lactone); ^1^H-NMR (CDCl_3_) δ 0.01 (s, Si(CH_3_)_2_), 0.77 (s, CH_3_-19), 0.89 (s, SiC(CH_3_)_3_), 0.97 (s, CH_3_-18), 0.62–2.00 (unresolved CH and CH_2_), 2.45 (m, CH_2_CO), 3.96 (t, *J* = 2.2 Hz, CH-3β); ^13^C-NMR (CDCl_3_) δ −4.85 (2×), 11.40, 14.45, 15.91, 18.12, 20.26, 23.78, 25.86 (3×), 27.91, 28.47, 29.50, 29.69, 31.87, 31.99, 32.44, 34.02, 35.92, 36.03, 36.69, 39.08, 47.04, 49.90, 54.22, 66.80, 93.55, 172.25.

#### Methylation of Lactone **9**

A solution of diisopropylamine (0.45 mL, 3.5 eq) in dry THF (2 mL) was stirred at 0 °C under an argon atmosphere and a 1.6 M solution of *n*-BuLi in hexanes (2.36 mL, 4.02 eq) was added dropwise. After 30 min, the resulting LDA solution was cooled at −78 °C and lactone **9** (0.445 g, 0.938 mmol) in dry THF (50 mL) was added. The mixture was allowed to stir 1 h at 0 °C and then cooled again at −78 °C before the addition of methyl iodide (4.02 mL, 6 eq) dropwise. The reaction mixture was stirred overnight from −78 °C to room temperature. Water was added to quench the reaction and the crude product was extracted with EtOAc. The organic phase was washed with a saturated NaCl solution, dried over MgSO_4_ and evaporated under reduced pressure. A column chromatography using a mixture of hexanes and EtOAc (9/1) as eluent allowed us to separate the three reaction products: the monomethylated lactone **10A**, the monomethylated lactone **10B** and the dimethylated lactone **11**, in proportions 2:2:1, respectively, in 70% yield.

*(3S,5S,8R,9S,10S,13S,14S,17R)-3-{[tert-Butyl(dimethyl)silyl]oxy}-5',10,13-trimethyloctadecahydrospiro[cyclopenta[a]phenanthrene-17,2'-pyran]-6'(3'H)-one* (**10**). **10A**: White solid; IR (film) υ 1729 (C=O, lactone); ^1^H-NMR (CDCl_3_) δ 0.01 (s, Si(CH_3_)_2_), 0.76 (s, CH_3_-19), 0.88 (s, SiC(CH_3_)_3_), 0.96 (s, CH_3_-18), 1.26 (d, *J* = 7.1 Hz, CH_3_-CH), 0.62–2.00 (unresolved CH and CH_2_), 2.38 (m, CHCO), 3.95 (t, *J* = 2.0 Hz, CH-3β); ^13^C-NMR (CDCl_3_) δ −4.86 (2×), 11.38, 14.52, 17.35, 18.08, 20.31, 23.65, 25.23, 25.85 (3×), 28.46, 28.56, 29.68, 31.85, 31.97, 32.43, 34.65, 35.93, 36.02, 36.12, 36.68, 39.07, 46.94, 49.80, 54.21, 66.78, 93.93, 175.06. **10B**: White solid; IR (film) υ 1729 (C=O, lactone); ^1^H-NMR (CDCl_3_) δ 0.004 (s, Si(CH_3_)_2_), 0.76 (s, CH_3_-19), 0.88 (s, SiC(CH_3_)_3_), 0.95 (s, CH_3_-18), 1.22 (d, *J* = 6.8 Hz, CH_3_-CH), 0.60–2.20 (unresolved CH and CH_2_), 2.52 (m, CHCO), 3.95 (t, *J* = 2.0 Hz, CH-3β); ^13^C-NMR (CDCl_3_) δ −4.88 (2×), 11.37, 14.49, 17.25, 18.06, 20.21, 23.87, 24.43, 25.84 (3×), 27.23, 28.45, 29.66, 31.84, 31.99, 32.33, 33.50, 34.02, 35.88, 35.99, 36.66, 39.02, 46.99, 49.63, 54.18, 66.77, 93.02, 176.01.

*(3S,5S,8R,9S,10S,13S,14S,17R)-3-{[tert-Butyl(dimethyl)silyl]oxy}-5',5',10,13-tetramethyloctadecahydrospiro[cyclopenta[a]phenanthrene-17,2'-pyran]-6'(3'H)-one* (**11**). White solid; IR (film) υ 1718 (C=O, lactone); ^1^H-NMR (CDCl_3_) δ 0.002 (s, Si(CH_3_)_2_), 0.76 (s, CH_3_-19), 0.87 (s, SiC(CH_3_)_3_), 0.95 (s, CH_3_-18), 1.23 and 1.25 (2s, 2 × CH_3_), 0.62–2.15 (unresolved CH and CH_2_), 3.94 (s, CH-3β); ^13^C-NMR (CDCl_3_) δ −4.88 (2×), 11.37, 14.54, 18.05, 20.29, 23.53, 25.52, 25.83 (3×), 27.61, 27.73, 28.44, 29.66, 31.58, 31.80, 31.99, 32.40, 34.82, 35.91, 36.00, 36.66, 37.71, 39.04, 47.01, 49.69, 54.17, 66.76, 93.91, 177.96.

#### Hydrolysis of the Silylated Ethers of **10A**, **10B** and **11**

Lactones **10A**, **10B** and **11** were respectively dissolved in dry THF. A 1 M solution of TBAF in THF (2 eq) was added and the resulting mixture was stirred overnight at refluxing temperature under an argon atmosphere. Water was then added and extraction was done with EtOAc. The organic phase was washed with a saturated NaCl solution and dried over MgSO_4_. The crude products were respectively submitted to Jones’ reagent as described above for the synthesis of **4**. A column chromatography using a mixture of hexanes and EtOAc (9/1) afforded the same mixture of monomethylated lactones **12**, in the case of **10A** and **10B**, and the dimethylated lactone **13** in the case of **11**.

*(5S,8R,9S,10S,13S,14S,17R)-5',10,13-Trimethylhexadecahydrospiro[cyclopenta[a]phenanthrene-17,2'-pyran]-3,6'(2H,3'H)-dione* (**12**). White solid; 90% yield; IR (film) υ 1714 (C=O, ketone and lactone); ^1^H-NMR (CDCl_3_) δ 0.99 (s, CH_3_-19), 1.03 (s, CH_3_-18), 1.23 and 1.27 (2d, *J* = 7.0 Hz and *J* = 7.2 Hz, CH_3_-CH in proportions 1/1), 0.62–2.60 (unresolved CH and CH_2_); ^13^C-NMR (CDCl_3_) δ 11.47, 14.52, (17.26), 17.35, 20.91, (20.98), (23.72), 23.94, (24.21), 24.41, 27.21, (28.58), 28.77, 31.44, 31.93, (33.50), 33.99, 34.64, 35.81 (2 ×), (36.15), 38.12, 38.60, 44.63, 46.75, 46.96, 49.45, (49.61), 53.67, 92.78, (93.65), 174.90, (175.89), 211.79; HRMS for C_24_H_37_O_3_ [M+H]^+^: calculated 373.2737, found 373.2739.

*(5S,8R,9S,10S,13S,14S,17R)-5',5',10,13-Tetramethylhexadecahydrospiro[cyclopenta[a]-phenanthrene-17,2'-pyran]-3,6'(2H,3'H)-dione* (**13**). White solid; 92% yield; IR (film) υ 1716 (C=O, ketone and lactone); ^1^H-NMR (CDCl_3_) δ 0.99 (s, CH_3_-19), 1.03 (s, CH_3_-18), 1.25 and 1.26 (2s, 2 × CH_3_), 0.63–2.50 (unresolved CH and CH_2_); ^13^C-NMR (CDCl_3_) δ 11.45, 14.56, 20.97, 23.61, 25.51, 27.65, 27.75, 28.75, 31.41, 31.55, 31.92, 34.77, 35.81, 37.76, 38.11, 38.58, 44.62, 46.74, 47.01, 49.49, 53.63, 93.70, 177.90, 211.84; HRMS for C_25_H_39_O_3_ [M+H]^+^: calculated 387.2894, 387.2900.

### 3.4. Synthesis of the Dispiro Compounds ***16*** and ***17*** (Scheme 3)

*(2R,5’S,8’R,9’S,10’S,13’S,14’S,17’S)-10',13'-Dimethylhexadecahydro-2'H-dispiro[oxirane-2,3'-cyclopenta[a]phenanthrene-17',2''-pyran]-6''(3''H)-one* (**14**). Trimethyl sulfoxonium iodide (1.3 g, 5.9 mmol) and sodium hydride 60% in mineral oil (236 mg, 5.9 mmol) was dissolved in DMSO (15 mL) and the mixture was stirred for 1 h at room temperature under an argon atmosphere. Compound **4** (0.5 g, 1.4 mmol) dissolved in THF (10 mL) was then added and the mixture stirred for 3 h. The reaction was quenched with a saturated solution of NH_4_Cl (35 mL) and the crude product was extracted with EtOAc and evaporated under reduced pressure. A column chromatography using a mixture of hexanes and EtOAc (8/2) afforded compound **14** (450 mg, 1.2 mmol) as a white solid in 72% yield. IR (film) υ 1720 (C=O, ketone and lactone); ^1^H-NMR (CDCl_3_) δ 0.86 (s, CH_3_-19), 0.98 (s, CH_3_-18), 0.70–2.15 (unresolved CH and CH_2_), 2.45 (m, CH_2_CO), 2.62 (s, CH_2_O); ^13^C-NMR (CDCl_3_) δ 11.26, 14.43, 15.84, 20.48, 23.78, 27.89, 28.38, 29.12, 29.45, 31.53, 31.96, 33.95, 35.51, 35.82, 35.85, 35,90, 43.72, 46.98, 49.79, 53.54, 53.84, 58.49, 93.42, 172.19.

*Methyl**-**N-{[(13S)-3-hydroxy-10,13-dimethyl-6'-oxoicosahydrospiro[cyclopenta[a]phenanthrene-17,2'-pyran]-3-yl]methyl}leucinate* (**15**). To a solution of the oxirane **14** (200 mg, 0.54 mmol) dissolved in MeOH (8 mL) was added L-leucine methyl ester (782 mg, 5.4 mmol) and the mixture was stirred in a Schlenck tube. After 22 h at 90 °C the mixture was dissolved in CH_2_Cl_2_ and concentrated under reduced pressure. The crude product was purified by a column chromatography using a mixture of hexanes and EtOAc (8/2) to give the amino alcohol **15** (276 mg) in 98% yield. IR (film) υ 3464 and 3333 (OH and NH); 1736 (C=O, ketone and lactone); ^1^H-NMR (CDCl_3_) δ 0.75 (s, CH_3_-19), 0.90 and 0.92 (2d, *J* = 6.6 Hz, 2 × CH_3_ from *i*-Pr), 0.96 (s, CH_3_-18), 0.70–1.98 (unresolved CH and CH_2_), 2.17 and 2.61 (2d of AB system, *J* = 11.9 Hz, CH_2_N), 2.43 (m, CH_2_CO), 3.22 (t, *J* = 7.3 Hz, CHC=O), 3.72 (s, CH_3_O); ^13^C-NMR (CDCl_3_) δ 11.23, 14.44, 15.86, 20.42, 21.94, 22.87, 23.79, 24.83, 27.90, 28.46, 29.47, 31.67, 31.71, 32.01, 33.80, 33.96, 35.91, 35.99, 38.52, 40.64, 42.77, 44.10, 47.01, 49.88, 51.71, 53.99, 59.25, 60.87, 69.88, 93.48, 172.24, 176.33.

*Methyl-(2S)-2-[(5R,5'S,8'R,9'S,10'S,13'S,14'S,17'S)-10',13'-dimethyl-2,6''-dioxooctadecahydro-2'H,3H-dispiro[1,3-oxazolidine-5,3'-cyclopenta[a]phenanthrene-17',2''-pyran]-3-yl]-4-methylpentanoate* (**16**). The amino alcohol **15** (69 mg, 0.13 mmol) was dissolved in CH_2_Cl_2_ (4 mL) and DIPEA (47.5 µL) was added. The solution was stirred for 10 min at 0 °C and triphosgene (20 mg, 0.07 mmol) was added in two portions to the mixture, which was stirred for 5 min at 0 °C and for 2.5 h at room temperature. Another portion of triphosgene (20 mg, 0.07 mmol) was then added and the mixture was stirred for 2 h at room temperature. A saturated solution of NaHCO_3_ was used to quench the reaction and the crude product was extracted with CH_2_Cl_2_. The organic phase was evaporated under reduced pressure and the crude product purified by column chromatography with hexanes and EtOAc (90/10 and 85/15) as eluent to give **16** (40 mg, 0.07 mmol) in 55% yield as a white solid. IR (film) υ 1740 (C=O, ketone, lactone and carbamate); ^1^H-NMR (CDCl_3_) δ 0.81 (s, CH_3_-19), 0.96 (s, CH_3_-18 and 2 × CH_3_ from *i*-Pr), 0.70–2.00 (unresolved CH and CH_2_), 2.44 (m, CH_2_CO), 3.13 and 3.42 (2d of AB system, *J* = 8.1 Hz, CH_2_N), 3.72 (s, CH_3_O), 4.59 (m, CHC=O); ^13^C-NMR (CDCl_3_) δ 11.40, 14.43, 15.84, 20.43, 21.06, 23.14, 23.76, 24.93, 27.91, 28.01, 29.47, 31.47, 31.93, 32.75, 33.87, 33.95, 35.31, 35.84, 37.69, 39.48, 40.86, 46.99, 49.77, 52.22, 52.89, 53.6 2×), 79.74, 93.40, 157.64, 171.99, 172.22; HRMS for C_32_H_50_NO_6_ [M+H]^+^: calculated 544.3633, found 544.3643.

(2R,5S,5'S,8'R,9'S,10'S,13'S,14'S,17'S)-10',13'-Dimethyl-5-(2-methylpropyl)hexadecahydro-2'H,6H-*dispiro[1,4-oxazinane-2,3'-cyclopenta[a]phenanthrene-17',2''-pyran]-6,6''(3''H)-dione* (**17**). To a solution of sodium methoxide (33 mg, 0.61 mmol) in dry THF (17.5 mL) was added the amino alcohol **15** (100 mg, 0.19 mmol) and the reaction mixture was stirred for 2 h at room temperature. The reaction was stopped by adding a saturated solution of NH_4_Cl and the crude product was extracted with EtOAc and purified by HPLC to generate the starting amino alcohol **15** (18%) and compound **17** (24 mg, 39%) as a white solid. IR (film) υ 1724 (C=O, lactone); ^1^H-NMR (CDCl_3_) δ 0.79 (s, CH_3_-19), 0.92 and 0.95 (2d, *J* = 6.2 Hz, 2 × CH_3_ from *i*-Pr), 0.96 (s, CH_3_-18), 0.75–2.02 (unresolved CH and CH_2_), 2.43 (m, CH_2_CO), 2.80 and 2.88 (2d of AB system, *J* = 13.5 Hz, CH_2_N), 3.50 (m, CHC=O); ^13^C-NMR (CDCl_3_) δ 11.36, 14.45, 15.84, 20.46, 23.40, 23.78, 24.46, 27.89, 28.00, 29.47, 31.38, 31.48, 31.96, 32.98, 33.92, 35.85, 36.02, 38.11, 39.29, 41.41, 46.99, 49.83, 52.41, 53.50, 55.58, 82.37, 93.42, 171.99, 172.25; HRMS C_30_H_48_NO_4_ [M+H]^+^: calculated 486.3578, found 486.3590.

### 3.5. Inhibition of 17β-HSD5

The enzymatic assay was performed using transfected (17β-HSD5) human embryonal kidney (HEK)-293 cells provided by Dr. Van Luu-The (CHUQ (CHUL)-Research Center) [29]. Briefly, 0.1 μM of the natural substrate [^14^C]-4-androstene-3,17-dione (Dupont Inc., Mississauga, ON, Canada) and 10 μL of an ethanolic solution of inhibitor were added to freshly changed culture medium in a 6-well culture plate containing HEK-293 cells overexpressing human 17β-HSD5. After incubation for 18 h, the reaction was stopped by adding a solution of unlabelled 4-androstene-3,17-dione (4-dione) and testosterone (T) before extracting twice with 2 mL of diethyl ether. The organic phase was pooled and evaporated to dryness. The metabolites were solubilised in dichloromethane, applied to silica gel 60 thin layer chromatography (TLC) plate (Merck, Darmstadt, GE), and then separated by migration in the toluene/acetone (4/1) solvent system. Substrates and metabolites were identified by comparing them to reference steroids, revealed by autoradiography, and quantified using the Phosphoimager system (Molecular Dynamics, Sunnyvale, CA, USA). The percentage of transformation (% *Transf*) and then the percentage of inhibition (% *Inh*) were calculated using the following equations: % *Transf* = 100 × [^14^C]-T (cpm)/([^14^C]-T (cpm) + [^14^C]-4-dione (cpm)) and % *Inh* = 100 × [% *Transf* (without inhibitor) − % *Transf* (with inhibitor)]/% *Transf* (without inhibitor). To avoid the enzyme inhibition by the resulting product of reaction (T), the quantity of enzyme (intact cells) and the incubation time were both selected to give a percentage of transformation below 30%, which is in a linear range.

### 3.6. Inhibition of 17β-HSD3 (Microsomal Fraction of Rat Testes)

A microsomal preparation of rat testes was obtained using slightly modified previously described procedures [30,31,32]. In brief, rat testes were homogenized on ice with a Polytron in cold phosphate buffer (20 mM KH_2_PO_4_, 0.25 M sucrose, 1 mM EDTA, pH 7.5) containing protease inhibitors mini-complete (Roche Diagnostics, Laval, QC, Canada) and centrifugated at 12,500g for 15 min to remove the mitochondria, plasma membranes, and cell fragments. The supernatant was further centrifugated at 100,000g for 45 min using an ultracentrifuge equipped with a 70.1 Ti rotor. The microsomal pellet was washed three times with phosphate buffer and centrifugated at 100,000g for 15 min. All these operations were conducted at 4 °C. The protein concentration of the supernatant was determined by the Bradford method using bovine serum albumin as standard [33]. The enzymatic assay was performed at 37 °C for 2 h in 1 mL of a solution containing 860 µL of 50 mM sodium phosphate buffer (pH 7.4, 20% glycerol and 1 mM EDTA), 100 µL of 5 mM NADPH in phosphate buffer, 10 µL of 5 µM [4-^14^C]-4-androstene-3,17-dione in ethanol (53.6 mCi/mmol, Perkin Elmer Life Sciences Inc., Boston, MA, USA), 10 µL of inhibitor dissolved in ethanol and 20 µL of diluted enzymatic source in phosphate buffer. Each inhibitor was assessed in triplicate. Afterwards, radiolabelled steroids were extracted from the reaction mixture with diethyl ether. The organic phases evaporated to dryness with nitrogen stream. Residue was dissolved in 50 µL of dichloromethane and dropped on silica gel 60 F_254_ thin layer chromatography plates (EMD Chemicals Inc., Gibbstown, NJ, USA) and eluted with a mixture of toluene/acetone (4:1) solvent system. Substrate ([^14^C]-4-dione) and metabolite ([^14^C]-T) were identified by comparison with reference steroids and quantified using the Storm 860 System (Molecular Dynamics, Sunnyvale, CA, USA). The percentage of transformation and then the percentage of inhibition were calculated as reported above (Section 3.5).

### 3.7. Proliferative and Antiproliferative Shionogi (AR^+^) Cell Assay

Assay for the proliferation of androgen-sensitive Shionogi mammary carcinoma cells as well as the inhibition of 0.3 nM DHT-induced proliferation was carried out according to the procedure described by Bydal and co-workers [11]. Calculations were performed according to the following equations and expressed as percentages: (a) Proliferative or androgenic activity = [(B − A)/(C − A)] × 100 and (b) Antiproliferative or antiandrogenic activity = [(C − D)/(C − A)] × 100, where A is the DNA content on cells incubated with control medium (μg), B is the DNA content of cells treated with the tested compound (μg), C is the DNA content of DHT-stimulated cells (μg) and D is the DNA content of DHT-stimulated cells treated with the tested compound (μg).

### 3.8. Steroids Receptor Binding Assays

The binding affinity assays on estrogen and progestin receptors from rat uterine tissue were carried out under the standard procedure established in our laboratory [34]. Assay for androgen receptor from rat ventral prostate was performed according to the procedure described by Luo and co-workers [35]. In the case of glucocorticoid receptors from rat liver tissue, the affinity binding assay was done using a slightly modified procedure described by Asselin and co-workers [36]. A dextran-coated charcoal adsorption, instead of a protamine sulfate precipitation, was used to achieve the separation of bound and free steroids.

## 4. Conclusions

Monospiro and dispiro steroid derivatives were efficiently synthesized from ADT or *epi*-ADT and characterized by IR, ^1^H-NMR, ^13^C-NMR and MS spectroscopies. Careful analysis of NMR data, especially ^13^C-NMR spectra, allowed the full assignment of all carbons for the series of monospiro and dispiro steroid derivatives. When tested as inhibitors of 17β-HSD5 and 17β-HSD3, the monospiro derivatives inhibited the enzyme according to the positioning and in accord with previously reported SAR studies. Thus, 17β-HSD5 was inhibited by the monospiro derivative at position C-17 whereas 17β-HSD3 was inhibited by a monospiro derivative at position C-3. For the first time, the presence of two spiro-functionalities was investigated as inhibitors of 17β-HSD3, but this strategy resulted in a lower inhibitory potency. Additional SAR results were generated for inhibiting 17β-HSD3 and 17β-HSD5, two key steroidogenic enzymes involved in biosynthesis of testosterone and in prostate cancer.
